# Dendritic Cells Regulate Treg-Th17 Axis in Obstructive Phase of Bile Duct Injury in Murine Biliary Atresia

**DOI:** 10.1371/journal.pone.0136214

**Published:** 2015-09-01

**Authors:** Yong-jun Liu, Kang Li, Li Yang, Shao-tao Tang, Xin-xing Wang, Guo-qing Cao, Shuai Li, Hai-yan Lei, Xi Zhang

**Affiliations:** Department of Pediatric Surgery, Union Hospital, Tongji Medical College, Huazhong University of Science and Technology, Wuhan, China; Texas A&M Health Science Center, UNITED STATES

## Abstract

Several cell types are considered to be effector cells in bile duct injury in rhesus rotavirus (RRV)-induced experimental biliary atresia (BA). Here, we identified an increased T helper 17 (Th17) cell population in a BA mode. By depleting the Th17 cells, the BA symptoms (onset of jaundice, acholic stools and retarded growth) were attenuated and the survival rate was improved. Furthermore, we found that in mice with BA, the percentage of CD4^+^CD25^high^Foxp3^+^ T regulatory (Treg) cells decreased along with the increased percentage of Th17 cells. However, the absolute numbers of Treg and Th17 cells were both increased in liver of RRV-injected mice compared to saline-injected mice. The proportion of Th17 cells at 7 days post-infection was decreased if Treg cells isolated from normal adult mice, but not Treg cells from the livers of mice with BA, were intraperitoneally transferred on day 5 of life. *In vitro* experiments also showed that Treg cells from mice with BA had a diminished suppressive effect on Th17 cell generation. To determine the mechanisms, we investigated the production of cytokines in the liver. The level of IL-6, which has been shown to be abundantly secreted by activated dendritic cells (DCs), was remarkably elevated. Importantly, in a Treg/Th17 cell suppression assay, IL-6 was demonstrated to paralyze the Treg cells’ suppressive effect on Th17 cells and eventually the unrestrained increase of Th17 cells contributed to bile duct injury. In conclusion, the DC-regulated Treg-Th17 axis, probably in conjunction with other effector T cells, aggravates progressive inflammatory injury at the time of ductal obstruction.

## Introduction

Biliary atresia (BA) consists of embryonic (fetal or prenatal; 20%) and perinatal (acquired; 80%) types, based on its clinical pathogenesis. The perinatal type is characterized by progressive inflammation, sclerosing cholangiopathy and obstruction of both the extrahepatic and the intrahepatic bile ducts. BA is the leading indication for liver transplantation in children. Recent studies have suggested that BA is an immune-mediated disease [1,2] and studies utilizing a rotavirus-induced BA mouse model have established further evidence for a virus-induced autoimmune pathway [3,4]. Experimental and clinical studies have proven that CD4^+^ T cells are implicated in the pathogenesis of BA, resulting in an augmentation of CD4^+^ T helper 1 (Th1) cells [5] and a depletion of T regulatory (Treg) cells [6]. However, the precise mechanisms by which the immune system is modulated in patients with BA remains unknown.

T helper 17 (Th17) cells are a unique subpopulation of CD4^+^ T cells [7] that express the transcription factor retinoic acid-related orphan receptor-γt (ROR-γt). Th17 cells induce a range of pro-inflammatory mediators that bridge the innate and adaptive immune responses, enabling the clearance of invading pathogens [8]. Although Th17 cells play a critical biological function in clearing extracellular pathogens, the inappropriate production of IL-17A by these cells is thought to be involved in the pathogenesis of various inflammatory, autoimmune diseases and transplant rejection in humans [9]. Others have linked Th17 cells to several autoimmune diseases of the liver. For example, IL-17A-deficient mice were found to be protected from multiple sclerosis [10]. Furthermore, IL-2Rα KO mice lost the repressive effect of IL-2 on Th17 cells, showed elevated levels of serum IL-17A, and finally suffered from primary biliary cirrhosis (PBC) [11]. Licata et al. found that in a well-established murine model of biliary obstruction by ligation, the bile ducts were infiltrated with populations of intrahepatic neutrophil and Th17 cells [12].

More recently, we have reported increased Th17 cell accumulation and a relative lack of Treg cells around the bile ducts and an involvement in cholangitis and bile duct damage in infants with BA [13]. We also found that a pro-inflammatory cytokine environment high in IL-1β and IL-6 in the liver promotes the continual differentiation and development of Th17 cells, but the precise mechanisms involved remain unclear.

The present study showed that Th17 cells are involved in the aberrant local immune response in experimental BA. We also investigated whether CD4^+^CD25^high^Foxp3^+^ Treg cells were capable of suppressing the differentiation and expansion of Th17 cells in the ductal areas in BA. We provide evidence that IL-6 secreted by hepatic activated dendritic cells (DCs) may have an impact on Treg/Th17 imbalance during neonatal bile duct obstruction.

## Materials and Methods

### Ethics statement

All animal experiments were approved by the Institutional Animal Care and Use Committee of Tongji Medical College, Wuhan, China (IACUC No. S358. Validity: 2011.02-2013. 12) and carried out in accordance with the guidelines of the Chinese Council on Animal Care in an AAALAC-accredited facility. All procedures were supervised by skilled veterinarians to ensure the animals’ welfare.

### Mouse model of BA

Wild-type Balb/c mice were kept in a specific pathogen-free, environmentally controlled facility and housed in a room with a 12-h dark-light cycle. Only the first litter of each mother was used in experiments. A total of 20μl (1.5 × 10^6^ PFU/ml) rhesus rotavirus (RRV; kindly provided as a gift by Prof. CL Mack, University of Colorado, Denver, USA) or 0.9% saline (for control mice) was injected intraperitoneally (i.p.) into Balb/c pups within the first 12 h of birth. Infected mice that died within the first 2 days were excluded from the study. Typical symptoms of BA in mice included oily fur, yellowish skin, acholic stool and retarded growth. Most infected mice, if untreated, would die within three weeks. Mice were sacrificed at 4, 7, 10 and 14 days after RRV or saline injection. Mouse whole liver/spleen samples were then pooled (n = 3–6 livers/pool); the results reflect ≥ 3 pools for all experiments. The mice were maintained on an irradiated sterile diet and provided autoclaved water. Before organs were harvested from mice, they were anesthetized and then humanely killed.

### Gene expression in mouse liver samples

Total RNA was extracted from frozen liver tissues from BA mice aged 4, 7, 10 or 14 days, using TRIzol (Invitrogen, Life Technologies Inc., Carlsbad, CA, USA) according to the manufacturer’s instructions. The extracted RNA was then subjected to quantitative real-time polymerase chain reaction (qPCR) using a Bio-Rad CFX96 qPCR instrument (Hercules, CA, USA). The expression of Foxp3, IL-17A and ROR-γt mRNA was normalized to GAPDH expression and relative gene expression was calculated using the 2-ΔΔCt method. The primer sequences for each gene are shown in S1 Table.

### Flow cytometry (FCM)

Mononuclear cells (MNCs) were isolated from livers harvested from saline- or RRV-injected mice on the 7th day post injection. The livers were gently minced in RPMI 1640 medium (HyClone, Logan, Utah, USA), passed through a 40-μm cell strainer (Millipore, Boston, Massachusetts, USA), and then centrifuged at 250 g for 10 min at 4°C. The supernatant was discarded and cells re-suspended in 33% Percoll (Sigma, St. Louis, MO, USA), followed by centrifugation at 400 g for 20 min at room temperature. The cells were then re-suspended in red-cell lysis buffer. After incubation for 5 min at 4°C, the cells were harvested by centrifugation and washed twice with phosphate-buffered saline containing 5% fetal calf serum (Life Technologies, Inc., Grand Island, NY, USA). Treg and Th17 cells were identified using the following antibodies (Abs) according to the manufacturer’s instructions: anti-CD4 FITC, anti-CD25 APC, anti-Foxp3 PE and anti-IL-17A PE (all from eBioscience, San Diego, CA, USA). The stained cells were analyzed with a FACS Calibur (BD Bioscience, Franklin Lakes, New Jersey, USA), and the data were analyzed using FlowJo Software 7.6.1 (Tree Star, Inc., Ashland, OR, USA).

### Fluorescent immunohistochemistry

Liver tissues removed from different groups were snap frozen in OCT (Miles Laboratories; Elkhart, IN) on dry ice. 6μm thick sections were stained. Slides were incubated with purified antibodies to CD4 (GTX44531, Gene Tex, Inc, CA, USA), IL-17A (Abcam, UK), and CK7(Abcam, UK) for 60 min at room temperature and counterstained with nuclear stain DAPI for 5 min (Aspin, China), then stained with the second antibodies conjugated with FITC(to show CD4), Alex Fluor 594 (to show IL-17A) and Alex Fluor 647 (to show CK7) for 1 hour at room temperature. The slides were sealed with Anti fluorescence quenching agent (Aspin, China) and visualized under 200× magnification with the Olympus IX 67 fluorescence microscopy (Olympus, Japan). Portal tracts were identified based on bile duct epithelial cells staining by CK7. Digital photographs were obtained with CellSens software (Olympus, Japan).

### Western blotting

The total protein content of the liver was boiled at 95°C for 5 min, cooled at room temperature for 5 min and centrifuged. Samples were run on 12% SDS-PAGE gels including a marker (MBI, Lithuania) for 2 h at 100 V and 4°C. The proteins were then transferred onto a NC membrane (Bio-Rad, Hercules, California, USA) in cold transfer buffer (10 mM Caps and 10% methanol, pH 11.0) under a constant current of 380 mA for 60 min at 4°C. Next, the blotted membranes were blocked and incubated with the appropriate diluted Ab (anti-Foxp3, 1:1000; anti-ROR-γt, 1:800; and anti-IL-17A, 1:800) (all from Abcam, Cambridge, UK). The membranes were subsequently incubated with a 1:4,000 dilution of a horseradish peroxidase-conjugated secondary Ab (Invitrogen, Logan, Utah, USA) for 1 h, followed by 3 washes with TBST for 15 min. The membranes were then processed using ECL (Bio-Rad, Hercules, California, USA) and exposed to film (Canon, Tokyo, Japan).

### Treatments of mice (intraperitoneal injection of digoxin, anti-IL-17A Ab or Treg cells)

To eliminate Th17 cells, 40 μg of digoxin (Sigma-Aldrich Co. LLC, Shanghai, China) per mouse was injected i.p. daily for 6 days, starting from 24 h post-RRV injection. Digoxin is known to deplete Th17 cells without affecting the differentiation of other T cell lineages [14].

To eliminate the effects of IL-17A, 20 μg of anti-IL-17A Ab (MAB421, R&D Systems, Minneapolis, MN, USA) was injected i.p. daily, starting immediately after RRV injection, for a total of 6 days. In control mice, an equal amount of rat IgG2A isotype control (MAB006, R&D Systems, Minneapolis, MN, USA) was used.

CD4+CD25^high^ Treg cells were purified using MicroBeads (Miltenyi Biotec, Bergisch Gladbach, Germany). The cell source was splenic MNCs isolated from 8-week-old adult Balb/c mice or 7-day-old BA mice using Ficoll according to the manufacturer’s protocols. FCM analysis confirmed that 90% of the live cells were CD4^+^CD25^high^ Treg cells. To confirm the regulatory effect of the Treg cells on Th17 cells *in vivo*, Treg cells (1.0 × 10^6^) suspended in saline were injected i.p. into RRV-infected neonatal mice on day 3 of life. Controls were subjected to the same protocol, except that the pups were injected with saline instead of Treg cells.

The stool color, weight gain and jaundice in the BA phenotype mice were monitored. Additionally, the numbers of Treg and Th17 cells in the liver were detected by FCM. Pathological sections of liver and gross views of the bile duct areas were also compared between the experimental and the control mice.

### DC isolation

For hepatic DC isolation, Balb/c (WT) mice were anaesthetized with isoflurane (induction 4%, maintenance 2%). After the portal vein was cannulated, the liver was perfused with cold RPMI 1640 containing 0.1% (w/v) collagenase D (Roche Diagnostics, Indianapolis, IN) before harvesting. Each liver was then gently minced and digested with 0.1% collagenase D in RPMI 1640 for 30 min at 37°C prior to passage through a 70-μm cell strainer, Percoll gradient centrifugation and separation with αCD11c+/αPDCA-1+ microbeads (Pan-DC Enrichment Kit, Miltenyi Biotec, Teterow, German).

### Th17 differentiation assay

Naïve CD4+ T cells (CD4+CD44lowCD62Lhigh) were purified using an EasySep Mouse Naïve CD4+ T Cell Isolation Kit (Stem Cell Technologies, Vancouver, British Columbia, Canada). The cell source was bulk CD4+ T cells isolated from splenic MNCs by negative selection according to the manufacturer’s instructions. The naïve CD4 cells were incubated in 12-well plates at 0.5 × 10^6^ in 2 ml of Th17-polarising medium containing 3 ng/ml TGF-β, 20 ng/ml IL-6, 5 ng/ml IL-1α, 10 μg/ml anti-IL-4, 10 μg/ml anti-IL-12, and 20 μg/ml anti-IFN-γ and stimulated with plate-bound anti-CD3 (OKT3; 10 μg/ml) and soluble anti-CD28 (2 μg/ml) monoclonal Abs (MAbs) for 7 days. In parallel groups, IL-6 was replaced with DCs from the livers of RRV-primed BA mice or the control group.

### Treg-cell suppression assay

To verify the suppressive effect of Treg cells from normal mice or BA mice on IL-17A production, we developed a system of co-culturing naïve CD4+ T cells, Treg cells, exogenous Th17-polarising cytokines and anti-CD3/CD28 Abs. Treg cells from either RRV-infected BA mice (n = 7) or saline-control mice (n = 8) at 2 weeks old were purified using a FACSAria cell sorter. In particular, Treg cells/naïve CD4^+^ T cells at a ratio of 1:4 to 1:16 were added to detect how Treg cells suppressed the process of naïve CD4^+^ T cells differentiating into Th17 cells (*vide supra*).

### Cytokine measurement and interventions

The concentrations of cytokines in the liver and spleen, including IL-2, IL-4, IL-5, IL-6, IL-10, IL-12, IL-23, IL-17A, IFN-γ, and TGF-β1, were measured with ELISA kits according to the manufacturer’s protocols (all kits were purchased from R&D Systems, Minneapolis, MN).

To determine the impact of the indicated cytokines on Treg/Th17 differentiation, purified naïve CD4^+^ T cells (0.5×10^6^) were cultured in 48-well plates in 1 ml of complete medium containing different cytokine combinations and were stimulated with plate-bound anti-CD3 and soluble anti-CD28 MAbs for 7 days. The percentages of Treg cells and Th17 cells were then measured by FCM.

### Statistical analyses

Animals were randomized using random number table into different groups. Results are presented as the mean value ± standard deviation (SD) and were compared using Student’s *t*-test. For experiments with more than 2 groups, statistical comparisons between all groups were performed using one-way analysis of variance, and further comparison between each of 2 groups were performed using Student’s *t*- test. The incidence of BA and the survival rate were compared using the Kaplan-Meier method in Fisher’s exact test.

## Results

### The proportions of Th17 cells are increased in EHBD of mice with BA

**Fig 1:**
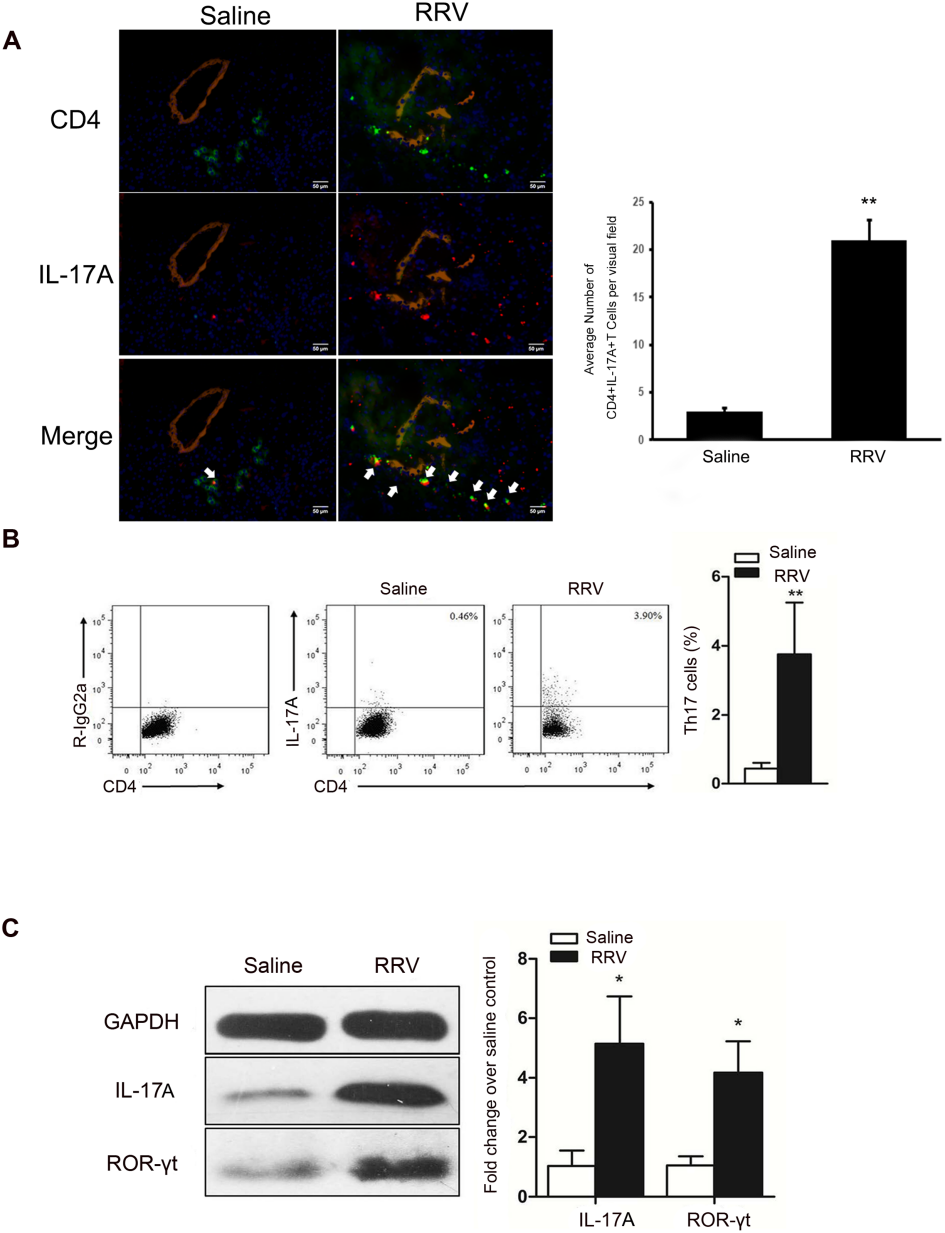
At the bile ductal obstructive phase, Th17 increased dramatically in the liver and was the main source of IL-17A. **(A)** Histopathological changes of the bile duct in RRV and saline groups on day 7 post infection. Antibodies to CD4 (Green) and IL-17A (Red) were added to distinguish CD4+ cells and IL-17A+ cells. Antibodies to Cytokine 7 (Khaki) indicate the bile duct lumen. Nucleus were stained by DAPI (Blue). The bile duct lumen was narrowed with a disrupted epithelium lining in BA mice. CD4^+^IL-17A^+^ T cells (Yellow, arrow indicate) together with other immunocytes infiltrated into the bile duct areas in BA mice. The magnification is x200. Representative of 4 experiments. * *P*<0.05. **(B)** Representative flow cytometry (FCM) diagrams of MNC in the liver of mice injected with saline or RRV. Th17 frequency is shown as a percentage of IL-17A+ T cells in liver CD4+ T cells. Leukocytes were purified using a Percoll gradient and cells were stained for CD4 and IL-17A, *P* = 0.001. **(C)** Representative western blots of IL-17A and ROR-γt in the saline and RRV group, GAPDH was used as the control. For IL-17A, *P* = 0.013, n = 3; for ROR-γt, *P* = 0.016, n = 3.

To determine whether Th17 cells have a role in biliary destruction during BA pathogenesis, we first showed that CD4^+^IL-17A^+^ T cells infiltrated into portal areas by immunofluorescence staining (Fig 1A). We found that at one week of age, the average number of CD4^+^IL-17A^+^ T cells per visual field was significantly increased in RRV-induced BA mice compared with saline-injected control mice (22.5 ± 1.49 vs 3.8 ± 1.7, P<0.01, Fig 1B). And then, we measured the number of Th17 cells present in the extrahepatic bile ducts (EHBD) of BA mice by flow cytometry. The percentage of CD4^+^IL-17^+^ T cells was significantly increased in RRV-induced BA mice compared with saline-injected control mice (3.76 ± 1.49% vs 0.44 ± 0.17%, P = 0.001, Fig 1B). Moreover, the protein expression of IL-17A and ROR-γt in the EHBD of BA mice was 5.1-fold and 4.2-fold higher, respectively, than in controls (Fig 1C).

### Depletion of Th17 cells induces decreased cellular infiltration in the liver and attenuated BA symptoms and improved survival

**Fig 2:**
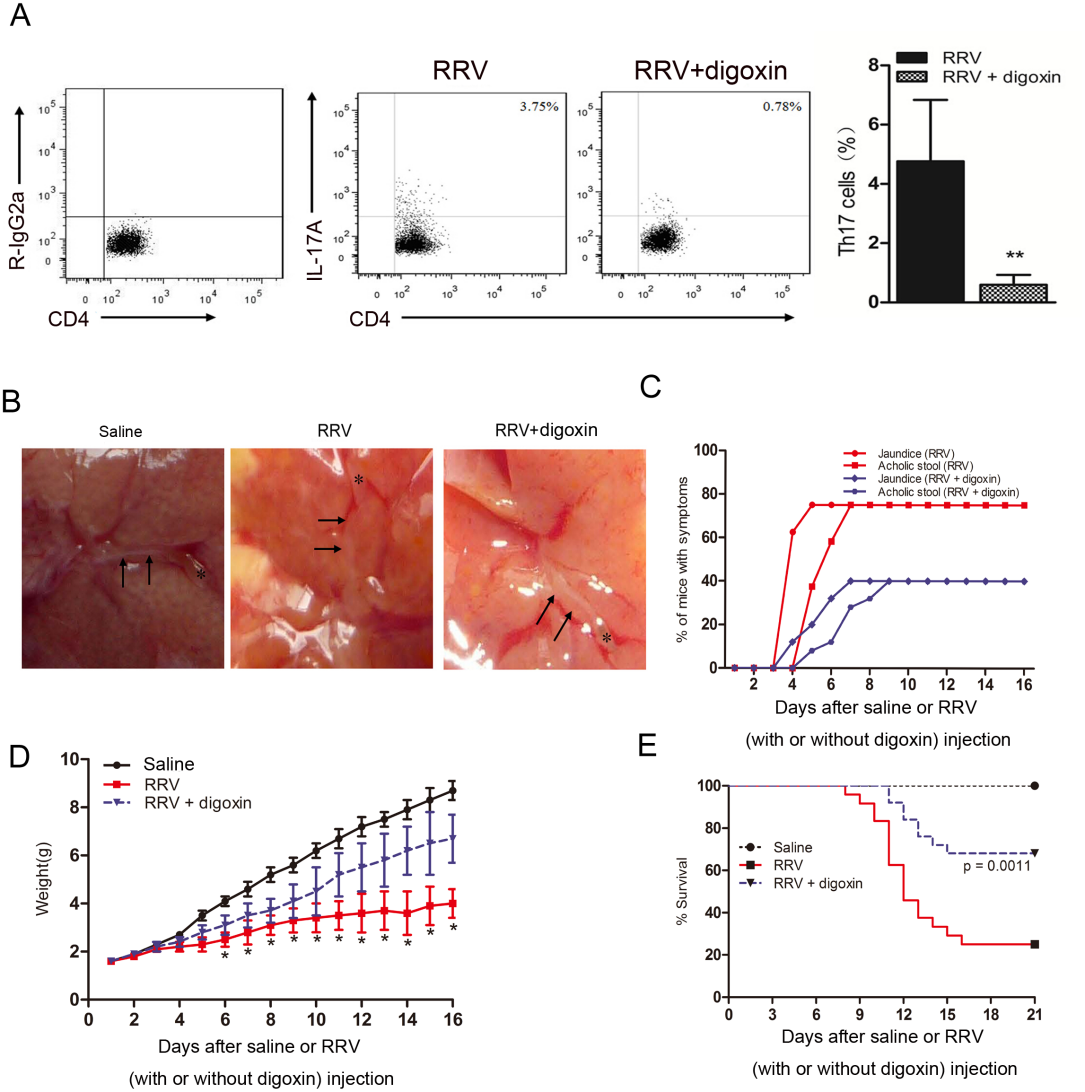
I.p injection of digoxin alleviates cellular infiltration and improves survival. **(A)** 40 μg digoxin was injected every day starting from 24 hrs post RRV infection for 6 days. The percentage of Th17 cells in the liver of mice 7 days post infection was measured by FCM. 87.6% of total Th17 in the RRV group was depleted by digoxin (4.76 ± 2.07% in RRV group *vs* 0.59 ± 0.33% in the RRV + digoxin group, *P* = 0.0074). **(B)** Morphology of extrahepatic (EHD) and intrahepatic bile ducts (IHD) obtained from mice at 7 days post RRV injection that had received either a digoxin or saline injection for 6 days. Pictures obtained by stereomicroscope, arrows highlight the bile duct epithelia. Representative of 4 experiments (x40). **(C)** Percentage of symptomatic mice in the two groups. Jaundice was judged by observation of skin areas without fur. Acholic stool was distinguished from a yellowish stool discharged by normal mice. **(D)** Weight gains after birth for 3 groups. Mice weights were recorded each day after RRV infection for a total of 16 days, * *P*<0.05. **(E)** Kaplan-Meier survival analysis of mice. 13–17 mice in each group were followed for survival post-infection. *P*<0.001 for RRV *v*s RRV + digoxin.

A total of 40 μg of digoxin was injected i.p. every day, starting from 24 h post-RRV infection. In the RRV group, 87.6% of the total Th17 cells were depleted by digoxin (Fig 2A). To explore the anatomical basis of the retardation of disease progression by Th17 cells, we next examined the effect of digoxin injection on the gross morphology and histological appearance of the hepatobiliary system. In saline-injected mice, the gallbladder was full with bile, and the lumen of the bile duct was patent. In the RRV-injected group, at day 7 after RRV injection, the gallbladder was small and contracted, whereas in the “RRV plus digoxin” group, an unobstructed bile duct and slight cholestasis were observed (Fig 2B). Injection of digoxin i.p. also caused attenuation of the BA phenotype, resulting in a decreased incidence of jaundice and acholic stool (Fig 2C). Furthermore, digoxin injection improved weight gain substantially and increased the median survival rate from 33.3% to 75.0% compared with that of age-matched RRV-infected mice (Fig 2D and 2E). Collectively, our data suggest that Th17 cells infiltrated the liver at the time of bile duct obstruction and that depletion of Th17 cells could improve survival, and reduce the possibility of bile duct obstruction-related symptoms in experimental BA.

Immunochemical staining revealed that the expression of IL-17A was decreased in the Th17-depleted group, but the level of IL-17A was still higher than that in the saline group (S1 Fig). Furthermore, beyond Th17 cells, we identified γδT cells as a potential source of IL-17A in the livers of experimental mice. Dynamic changes in the percentages of these two cell subgroups from day 1 to day 14 post-injection in the RRV and saline groups were measured by FCM. The number of Th17 cells increased 200-fold at 10 days compared to day 1 after the RRV challenge. In contrast, the γδT cell number decreased gradually after RRV challenge (S2 Fig). When anti-IL-17A Ab was injected i.p. into mice daily for 6 days, beginning from 24 h post-RRV infection, compared with RRV-primed mice without anti-IL-17A Ab injection, the survival rate was increased and weight gain was improved (S3 Fig).

### The percentage of Treg cells decreases, but the absolute number of Treg cells increases in BA mice in the obstructive phase

**Fig 3:**
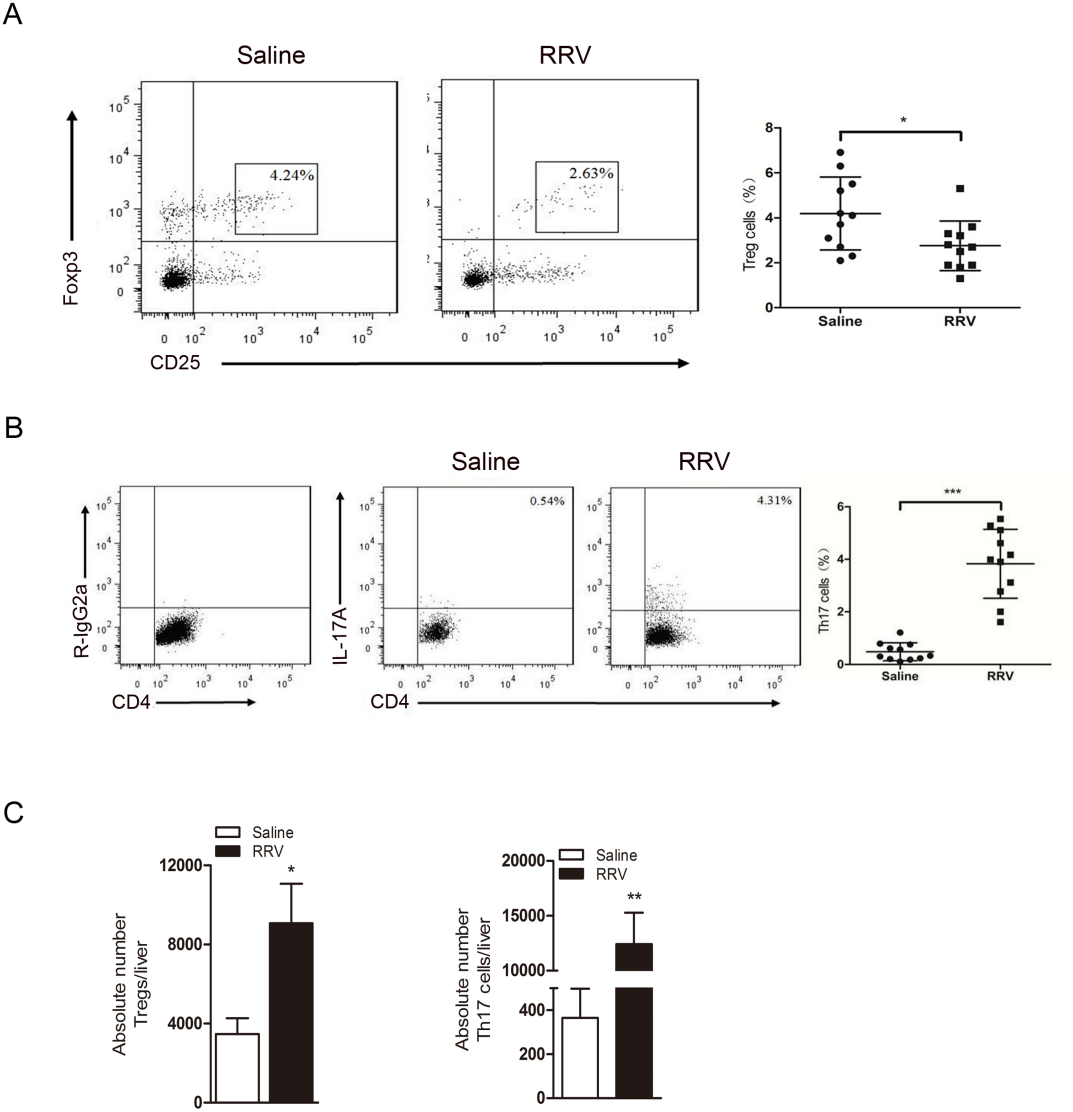
The proportion of Treg decreases and the proportion of Th17 cells increases, but the absolute numbers of both are increased. **(A and B)** At the time of bile ductal obstruction, flow cytometry analysis showed that the percentage of Tregs decreased but Th17 increased in the liver of the RRV group compared to the saline group. The percentage of CD4^+^ cells positive for the indicated marker is shown. *P* = 0.0245 for the Treg group and *P*<0.001 for the Th17 group, n = 11 for each group. **(C)** Absolute numbers of Tregs increased 2.6 fold and Th17 increased 34 fold in the RRV challenged group compared to the saline control group. Numbers were measured per individual liver by FCM, * *P*<0.05, ** *P*<0.01, n = 5 for each group.

In the present study, Treg cells were defined by expression of the surface markers CD4 and CD25 and the transcription factor Foxp3 (CD4^+^CD25^high^Foxp3^+^). The majority of this subset was CD127^-^ (96–98%, data not shown). We found that the Treg cell proportion decreased in livers at the 7th day post-RRV injection compared with the proportion in saline-injected mice (4.19 ± 1.62% *vs* 2.75 ± 1.11%, *P* = 0.025, n = 11, Fig 3A). In contrast, the percentage of Th17 cells increased 8.0-fold (0.48 ± 0.34% *vs* 3.83 ± 1.31%, n = 11, *P*<0.000, Fig 3B). However, the absolute numbers of Treg cells and Th17 cells increased 2.6-fold (3,477 ± 793/liver in the saline group *vs* 9,075 ± 1,998/liver in the RRV group, *P* = 0.011) and 34-fold (365 ± 133/liver in the saline group *vs* 12,410 ± 2,887/liver in the RRV group, *P* = 0.002), respectively (Fig 3C).

### Adoptive transfer (AT) of normal Treg cells suppresses RRV-induced generation of Th17 cells

**Fig 4:**
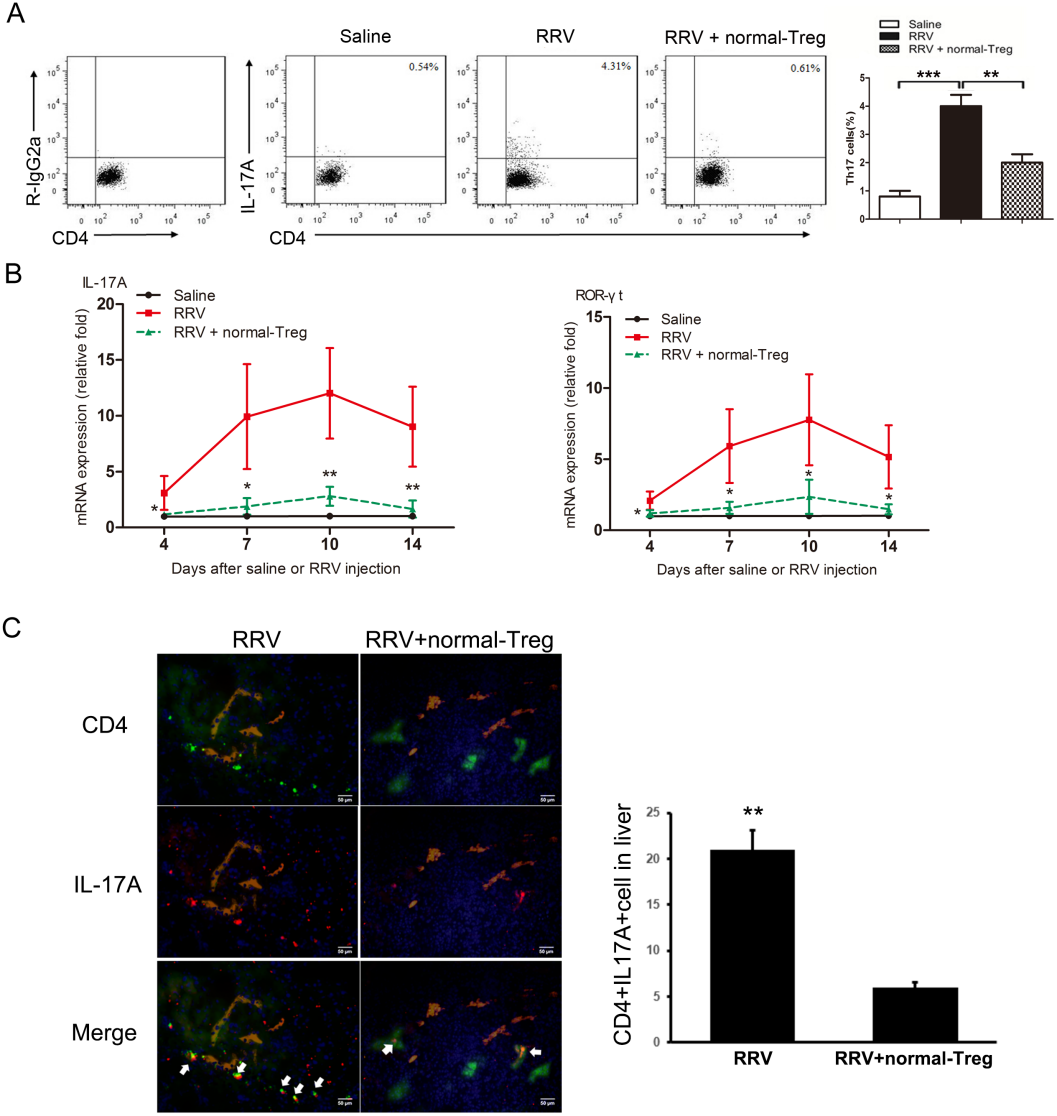
Adoptive transfer of normal Treg cells into BA mice suppresses RRV-induced generation of Th17 cells. Saline or RRV was injected within 12 hrs of birth. Tregs were injected i.p. 3 days post infection. **(A)** Representative FCM diagrams of Th17 cells from liver of RRV primed 7 days old mice. *** *P*<0.001, ** *P*<0.01, n = 11. **(B)** Hepatic mRNA for genes encoding IL-17A and its transcription factor ROR-γt were quantified by real-time PCR and expressed as fold change (vertical axis) in mice with BA over controls, n = 9 for biliary atresia and n = 7 for controls; all fold changes were statistically significant at * *P*<0.05, ** *P*<0.01. **(C)** Immunofluorescence staining of liver frozen sections of mice at day 7 post infection. Antibodies to CD4 (Green) and IL-17A (Red) were added to distinguish CD4+ cells and IL-17A+ cells. Antibodies to Cytokine 7 (Khaki) indicate the bile duct lumen. Nucleus were stained by DAPI (Blue). Magnification x200. * *P*<0.05. Representative of 4 experiments.

Different studies have shown that AT of normal Treg or of CD4^+^ T cells containing Treg cells can restrain the inflammatory response to a viral challenge. Based on this, we hypothesized that Treg cells may also suppress the generation of Th17 cells. To explore the possibility that Treg cells may suppress Th17 differentiation *in vivo*, normal splenic Treg cells were purified and transferred into mice at 3 days post-RRV infection where the Th17 cells began to increase rapidly (S2 Fig). Following AT, the frequency of Th17 cells was significantly reduced at day 7 post-infection (Fig 4A). This decreased frequency was associated with downregulation of the hepatic expression of IL-17A and ROR-γt mRNA in these mice compared with mice without AT (Fig 4B). Immunofluorescence staining further demonstrated that the expression of IL-17A and ROR-γt was reduced around bile duct areas after AT (Fig 4C).

Collectively, these data demonstrated that overexpansion of Th17 cells in BA at the time of bile duct obstruction could be constrained by transferring normal Treg cells. Partly by downregulating Th17 cells, AT of Treg cells alleviated ductal inflammation and improved survival.

### The ability of Treg cells to suppress Th17 cells is impaired in mice with BA

**Fig 5:**
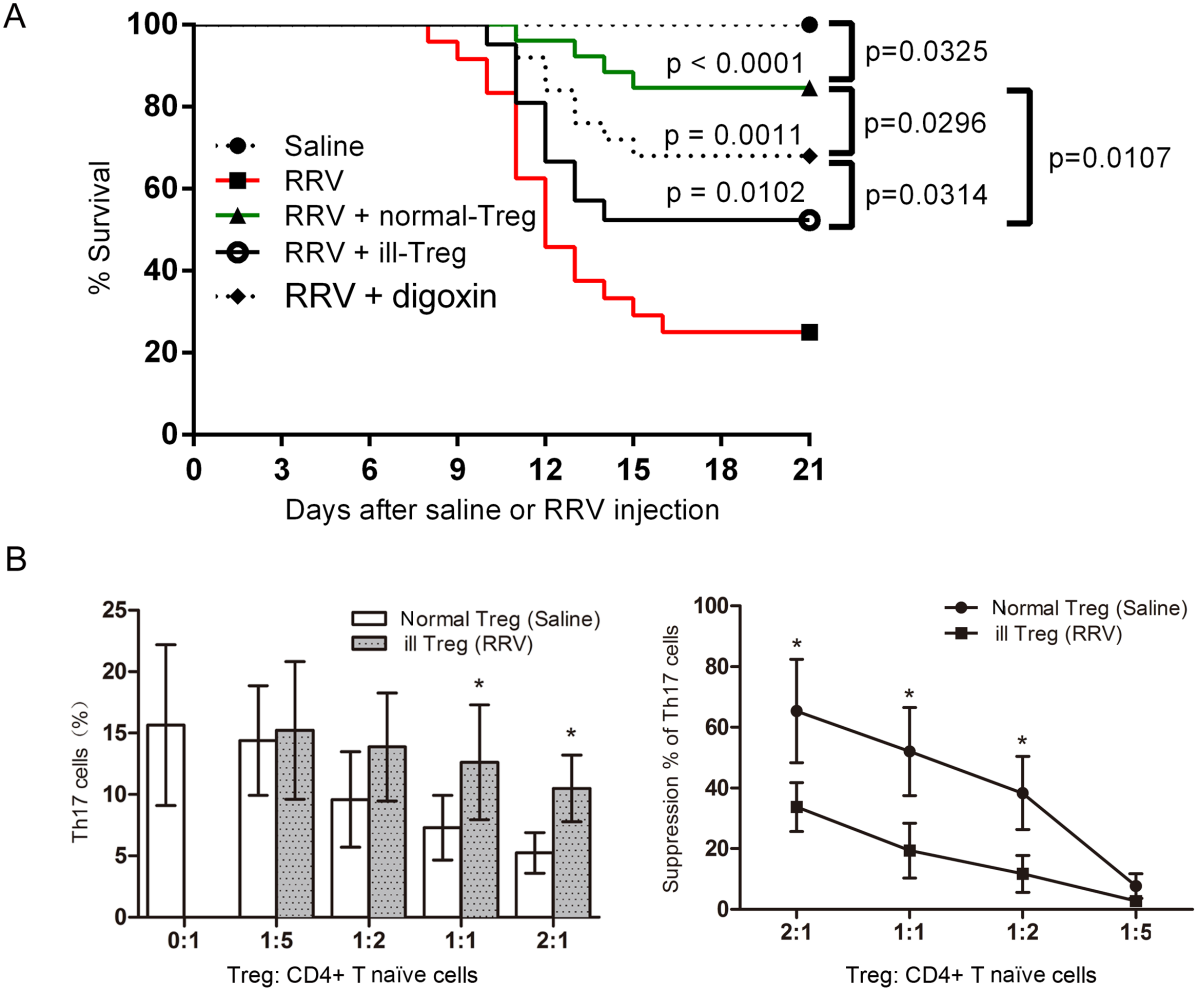
Suppression capability of “ill” Tregs on the proliferation of Th17 originating from CD4+ naïve T cells *ex vivo*. **(A)** Tregs isolated from BA mice were injected i.p. 3 days post infection. The survival rate of mice that received Tregs from BA mice was significantly lower than mice that received Tregs from normal mice (*P* = 0.0107). **(B)** CD4+ naïve T cells were isolated from splenic MNCs of 8-week-old adult Balb/c mice. Tregs were obtained from splenic MNC of either RRV or saline primed 7 days old mice. Different ratio of CD4+ naïve T cell and Tregs were cultured in the conditions described above (TGF-β, IL-6, IL-1α, anti-IL-4, anti-IL-12, anti-IFN-γ, and stimulated with plate-bound anti-CD3and soluble anti-CD28 MAbs for 7 days). IL-17A in the supernatant was measured by ELISA. The percentage of relative suppression was calculated by the formula: 1-(IL-17A in the presence of Treg)/(IL-17A in the absence of Treg). 10–12 pooled spleens were used in experiments. * *P*<0.05.

Despite the fact that the absolute number of Treg cells was increased, the proliferation of Th17 cells and the inflammation of bile ducts were not suppressed in BA mice. Therefore, we suspected that the ability of Treg cells to suppress Th17 cells was impaired in BA. To determine directly the suppressive effect of the Treg cells on Th17 cells, Treg cells isolated from 7-day-old BA mice were injected i.p. into RRV-primed mice at 3 days post-infection. These Treg cells were isolated from the spleen (which is a natural reservoir for Treg cells) with similar Treg cell responses that were observed in the liver following RRV infection. The survival rate of this group of mice was 52.4%, which was significantly decreased compared with the survival rate of mice after AT of normal Treg cells (84.6%, *P* = 0.011, Fig 5A).

In an *ex vivo* experiment, Treg cells were co-cultured with naïve CD4^+^ T cells from age-matched normal mice in the presence of TGF-β and IL-6. The Treg cells had been purified from 7-day-old BA mice or from normal mice, which served as controls. Without AT of Treg cells, IL-17A production in the supernatant was observed to be 16 pg/ml (Fig 5B). This concentration decreased as the number of Treg cells from control mice was increased. However, Treg cells isolated from BA mice were not as efficient at inhibiting the production of IL-17A in the presence of the same stimulation with anti-CD3 and anti-CD8; in particular, the suppression rates were decreased compared with the rates for Treg cells from normal mice across all cell ratios tested.

### Impact of cytokines on Th17 cells and Treg cells in BA mice

**Fig 6:**
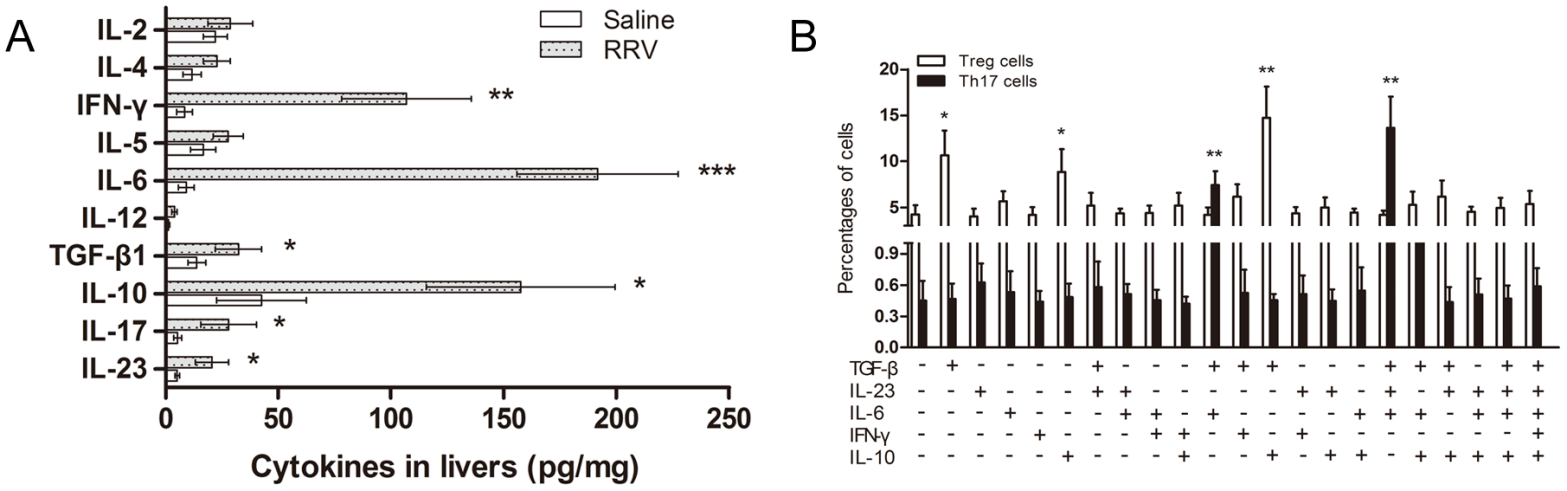
Impact of cytokines on Th17 and Treg cells in BA mice. **(A)** Cytokine levels in the supernatant of liver cell suspensions of RRV or saline injected mice were determined by ELISA and are reported as the mean ± SEM (n = 15). * *P*<0.05, ** *P*<0.01, *** *P*<0.001. **(B)** Th17 cells detected in culture medium of CD4+ naïve T cells stimulated with plate-bound anti-CD3 and soluble anti-CD28 mAbs in the presence or absence of the indicated cytokines, followed by re-stimulation with PMA and ionomycin 7 days later. The results are reported as the mean ± SEM (n = 10). * *P*<0.05, ** *P*<0.01.

We detected certain cytokines that are probably related to the Th17-Treg imbalance when examining the livers of BA mice. As shown in Fig 6A, significantly higher levels of IFN-γ, IL-6, IL-10, TGF-β and IL-23 were found in the livers of BA mice compared with controls (*P*<0.05). To evaluate effects of these cytokines on the generation and differentiation of Th17 and Treg cells, purified naïve T cells were cultured in the presence of one or more cytokine milieus. We noted that only TGF-β increased the Treg cell population. The rest of the cytokine identified above, even when highly expressed, did not increase the percentage of Th17 cells, whereas a combination of IL-6 and TGF-β, or IL-6, TGF-β and IL-23, markedly increased the percentage of Th17 cells (Fig 6B). Despite the fact that TNF and IL-10 levels were significantly increased in the livers of BA mice, these cytokines seemed to be not important for Th17 differentiation. The results suggest that IL-6, the cytokine whose levels were markedly increased in the livers of BA mice, helps to polarize the local microenvironment for the production of Th17 cells, but not Treg cells.

### IL-6 produced by activated DCs promote Th17 differentiation *ex vivo*

Several reports have noted that DCs are persistent in the liver as biliary injury progresses and are cellular targets of RRV in early postnatal life in BA. Based on the ability of DCs to produce cytokines following stimulation, we tested the ability of DCs to produce IL-6 in the obstructive phase *ex vivo*. Because IL-6 can also be secreted by many cell types other than DCs, we tested IL-6 levels in the culture medium of macrophages, B cells, CD4^+^CD25^-^ T cells and cholangiocytes isolated from the livers of BA and control mice (S4 Fig). The results showed that DCs produced the highest IL-6 levels and highest fold changes in all cell types listed above in the BA mice compared with control mice.

**Fig 7:**
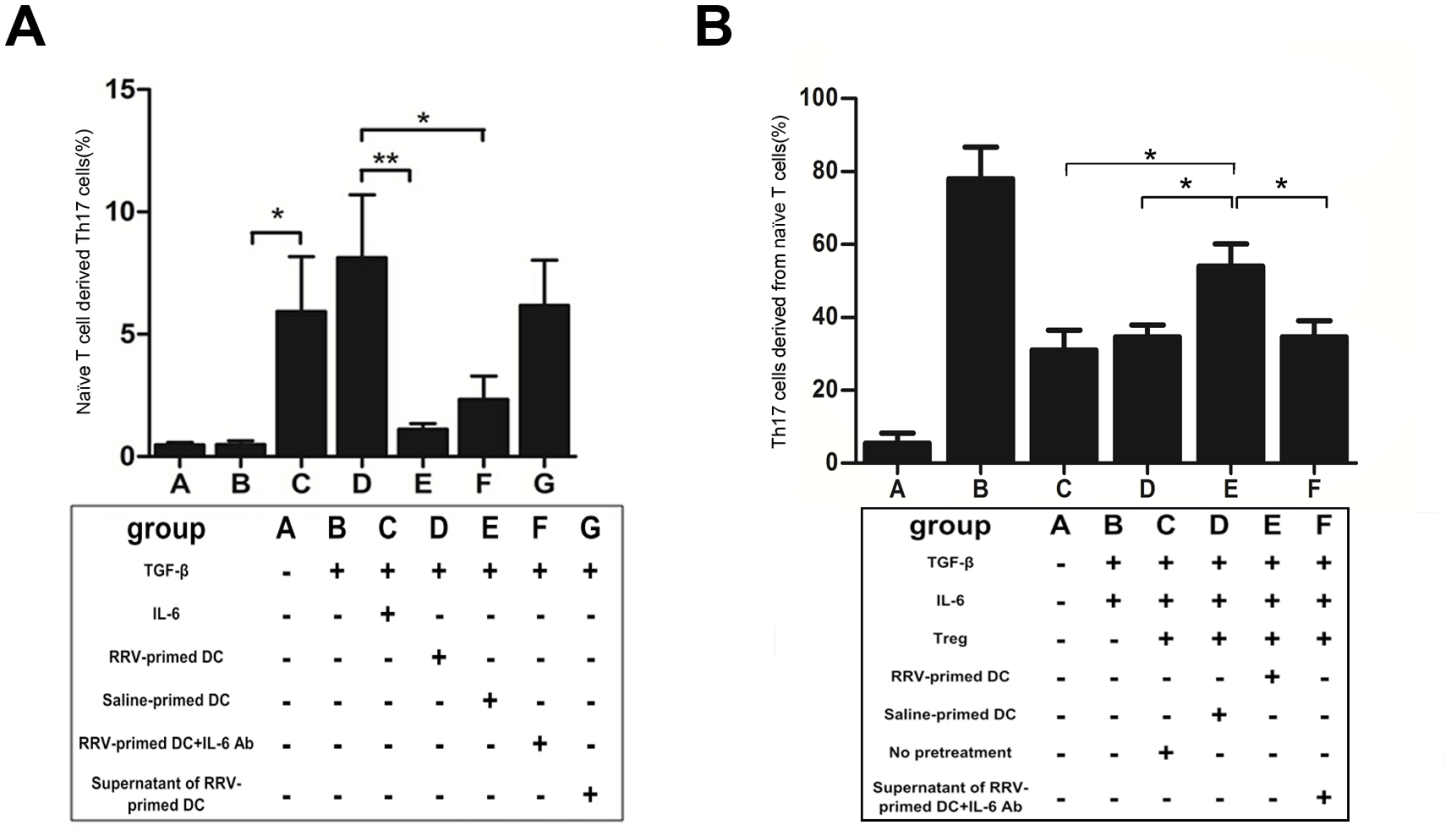
Activated DC produces abundant IL-6, hinders the Treg suppression function and promotes Th17 generation *ex vivo*. **(A)** DCs were isolated from mice liver on the 7^th^ day after injection of RRV or saline and within 12 hrs of birth. DCs were added to the differentiation assay containing 3 ng/ml TGF-β, 20 ng/ml IL-6 and the other indicated cytokines, and a stimulus (*vide supra*). After 7 days, the percentage of Th17 was measured by FCM and the data is reported as the mean ± SEM (n = 12). * *P*<0.05, ** *P*<0.01. **(B)** Tregs were isolated from liver and spleen of mice on the 7^th^ day after injection of RRV or saline within 12 hrs of birth. Different groups of Tregs were pretreated by co-culturing with DCs obtained from the liver of either RRV or saline injected mice, before adding them to the suppression assay as described above. In group F, Tregs were pretreated with supernatant from RRV-primed DC + IL-6 Ab. The production of IL-17A in the supernatant was measured by ELISA and is reported as the mean ± SEM (n = 11). * *P*<0.05, ** *P*<0.01.

To address the physiological role of IL-6, we established a modified Th17 differentiation assay. In this assay, adding an excess of IL-6 (30 ng/ml) markedly increased the percentage of Th17 cells in the culture system containing naïve CD4^+^ T cells and TGF-β, together with the indicated cytokines and stimulus (*P* = 0.005, Fig 7A, column C *vs* B). When we added DCs (or their supernatant) from RRV-primed mice instead of IL-6, the percentage of Th17 cells was maintained (*P* = 0.408, Fig 7A, groups D and G compared with group C). In contrast, when DCs from saline-injected rather than RRV-injected mice were added, the Th17 cell percentage dropped to 1.1% (*P* = 0.012, Fig 7A, column E *vs* D). If RRV-primed DCs were added together with 20mg anti-IL-6 Ab (ab6672, Abcam, Cambridge, UK), Th17 cell production was also significantly decreased, comparable to what was observed for RRV-primed DCs alone (2.34 ± 0.94% *vs* 8.13 ± 2.56%, *P*<0.01, Fig 7A, column F *vs* D). These data indicate that the soluble cytokine IL-6, secreted by RRV-activated DCs rather than inactivated DCs, plays a vital role in Th17 differentiation.

### IL-6 produced by DCs renders Treg cells incapable to suppress IL-17A production

Our *in vivo* experiment showed that even the absolute number of Treg cells was increased after RRV challenge, but these cells were incapable of suppressing the generation of pro-inflammatory Th17 cells. This impaired suppressive effect of Treg cells was shown in both *in vivo* and *ex vivo* experiments, but the mechanism was not determined. In addition, the level of IL-6 was robustly increased in the inflamed liver and contributed to the induction of Th17 differentiation. Thus, we hypothesized that the high level of IL-6 contributed to sequestration of Treg cells. To test this hypothesis, we pretreated Treg cells by incubating them for 36 h with anti-CD3 and the supernatant of DCs isolated from RRV-infected BA mice or control mice, and then added the Treg cells to a Th17-cell suppression assay. The efficacy of suppression was measured as described above. The results showed that Treg cells pretreated with primed DCs from RRV-injected mice were not as efficient in suppressing IL-17A production as untreated Treg cells, or Treg cells pretreated with DCs from saline-primed mice (4.73 ± 0.86% *vs* 2.67 ± 0.64%, *P* = 0.029, Fig 7A, column E *vs* C/D). This inefficiency of suppression was reversed when 20 mg anti-IL-6 Ab was added to the supernatant of cultured DCs isolated from the livers of RRV-primed BA mice (2.83 ± 0.22% *vs* 7.62 ± 0.43%, *P* = 0.009, Fig 7B, column F *vs* E).

## Discussion

We previously demonstrated that Th17 cell numbers were increased and IL-17A levels were elevated in the liver of infants with BA [13]. However, the underlying mechanism is unknown. In the present study, we identified an increased population of Th17 cells in the livers of mice exposed to RRV soon after birth in an experimental model of BA. The pro-inflammatory property of Th17 cells was verified by the fact that digoxin (an inhibitor of ROR-γt) and anti-IL-17A Ab could attenuate BA symptoms and improve prognosis. During complementary *ex vivo* and *in vivo* experiments, we demonstrated that the Th17 subgroup could be regulated by Treg cells. Furthermore, we found robustly increased IL-6 levels secreted by activated DCs in the liver, which was shown to favor the production of Th17 cells rather than Treg cells in two ways. First, together with TGF-β, IL-6 promoted Th17 differentiation. Second, IL-6 hindered the Treg cells suppressive effect on Th17 cells. Eventually, the unrestrained increase in Th17 cells contributed to bile duct injury. The DC-regulated Treg-Th17 axis, probably in conjunction with other effector T cells, aggravates progressive inflammatory injury at the time of ductal obstruction.

Several cell types participate in bile duct injury in experimental BA induced by RRV infection in mice. For example, Tucker and others have demonstrated that BA mice had increased numbers of T cells producing IFN-γ and TNF-α [5,15]. In addition, *in situ* hybridization of human liver sections revealed that Th1 cytokine-producing cells were located in the portal tracts in BA [16]. However, Stat1^-/-^ mice, which lack the ability to mount Th1 immune responses, could also display duct epithelial injury and a BA phenotype after RRV infection, which was shown to be mediated by a prominent Th2 response [17]. In addition, Ly6C+CD44+ effector CD8 cells were also considered as a subset of T lymphocytes in Balb/c mice with RRV-induced BA, with enhanced cytotoxic killing and IFN-γ production [18]. Other evidence has shown that activated NK cells target the mouse duct epithelium and drive tissue-specific injury at the time of obstruction [6,19].

Here, our study clearly showed that, other than the Th1 and Th2 subgroups, a subgroup of IL-17A-producing effector T helper cells, called Th17 cells, was increased in the livers of RRV-induced experimental BA mice. It has been reported that Th17 cells exhibit pro-inflammatory properties by producing IL-17A, a cytokine that acts on a broad range of cell types to induce the expression of other cytokines, including TNF, IL-1β, GM-CSF, chemokines and metalloproteinases [20,21]. In addition, Th17 produces IL-22, TNF-α, IFN-γ, CCR6 and CCL20 (a ligand for CCR6), which recruit other immune cells [22]. To support the hypothesis that Th17 cells are actually involved in the pathogenesis of BA and are not just a reflection of immune activation, we i.p. injected the ROR-γt inhibitor digoxin to deplete Th17 cells in a mouse model of BA. Attenuated BA symptoms and improved survival were observed in these mice, indicating that Th17 cells may be involved in the substantial inflammatory response to RRV challenge.

IL-17A is the founding member of the IL-17 family of cytokines, which includes IL-17A (also called IL-17), IL-17B, IL-17C, IL-17D, IL-17E (produced by Th2 cells), and IL-17F [22]. Both IL-17A and IL-17F are reported to be involved in multiple human autoimmune diseases, but their functions and potency may vary. IL-17A antibody helped to improve survival in BA model, which confirmed the pathogenic role of IL-17A in BA model. To rule out the possibility that antibody treatment in mice increase survival by depletion of virus infected cells, we evaluated RRV gene VP6 expression by qPCR relative to GAPDH in IL-17A antibody treatment group compared to RRV infected mice without IL-17A antibody treatment. Similar RRV replication level was seen in two groups (S5 Fig).

We also explored IL-17F mRNA level in RRV challenged mice, as well as in mice of Th17 depleted experiment and Treg adoptive transfer experiment. The mRNA level of IL-17F is not significantly changed in RRV challenged mice, but it did decrease in digoxin treated mice and Treg transferred mice (S6 Fig). In future studies, IL-17A knockout mice and IL-17F knockout mice might be needed to confirm the separate role of IL-17A/F in BA pathogenesis.

In diseases other than BA, IL-17A and IL-17F have been reported to be produced not only by activated Th17 cells but also by several other cell types in innate immunity, and especially γδT cells [23,24]. In the present study, we found low numbers of γδT cells within livers affected by BA in the first week after birth, with a slightly declining tendency over time. Thus, in the bile duct obstruction stage, IL-17A is probably predominantly produced by activated Th17 cells, rather than γδT cells.

The depletion of Treg cells early after perinatal RRV infection has been implicated in the pathogenesis of BA in murine models [6,15]. This concept is further supported by the fact that if Treg cells were depleted in older mice, the expansion of effector lymphocytes in the liver would dramatically aggravate hepatobiliary injury [18]. In infants with BA, deficiency of Treg cells has also been observed at the time of diagnosis [25]. Interestingly, the results of our study showed that at 7 days after RRV injection, despite the diminished percentage of Treg cells among the total CD4^+^ T cells, the total number of Treg cells was increased compared with the number in saline-injected controls. However, the existence of an overwhelming abundance of Th17 cells in the liver suggests that the increased quantity of Treg cells was still inefficient in suppressing Th17 cells at the time of ductal obstruction. The impaired suppressive ability of Treg cells, as observed by our group and others, is not able to restrict the infiltration of effector T cells, including Th17 cells, fostering inflammatory damage to the bile ducts.

Along with this Treg-Th17 imbalance, we also detected the cytokine profile in the pro-inflammatory microenvironment of BA livers, which was characterized by markedly increased levels of IL-6 and other cytokines. To address the physiological roles of the cytokines, we examined the effect of different cytokine combinations in the Th17 differentiation assay. The results indicated that IL-6 in concert with TGF-β polarized the local milieu to favor the production of Th17 cells rather than Treg cells, which is in keeping with reports by other investigators on liver diseases other than BA [26]. Moreover, Treg cells pretreated with IL-6 were sequestered, preventing suppression of Th17 cells in *ex vivo* experiments. Taking the results together as a possible explanation, IL-6 has a dual role as an inducer of Th17 differentiation and as an inhibitor of spontaneous Treg cell activation. Another mechanism might be related to the possibility that in the cytokine milieu of extensive IL-6 expression, Foxp3^+^ Treg cells could express the ROR-γt gene and secrete IL-17A [27]. We indeed identified double-positive IL-17A^+^Foxp3^+^ T cells in the liver (data not shown).

Notably, a large amount of IL-6 was produced by DCs in BA mice, which was consistent with other reports [28]. This finding is not surprising because in RRV-challenged mice, DCs are persistently anchored in the liver as biliary injury progresses [29]. Working as an important antigen-presenting cell (APC) in innate immunity, the activated DCs in locally inflamed livers have been described to interact with various cell types in other studies [6,18,19,29]. Based on our data, by secreting IL-6, DCs bridge the innate and adaptive immune systems as the mouse mounts a response to clear infected cells, resulting in the atresia phenotype as a secondary event.

The difference in IL-6 levels between the livers of BA mice and age-matched normal mice provide an insight into the potential mechanism of susceptibility to biliary injury in the first 3 days of life. A similar period of susceptibility is recapitulated in humans, in which the onset of disease is restricted to the first 3–4 weeks after birth. Other studies have described Foxp3^+^ natural Treg cells as actively inhibiting the maturation of DCs [30]. However, in the current study, the paucity of Treg cells in the early neonatal period was incapable of inhibiting DC activation, resulting in excessive IL-6 secretion by RRV-stimulated DCs. The high level of IL-6 in turn partially paralyzed Treg cells, induced overproduction of pro-inflammatory Th17 cells and aggravated bile duct injury.

The present study focused mainly on immune regulation during ductal obstruction at 7 days post-infection, which was considered to be the peak of inflammation of the bile duct. The networks of immune cell involvement and crosstalk at different stages of the immune response are complicated. For instance, Treg cells might suppress NK cell activation or pDC co-stimulation at an earlier stage of bile duct injury, as reported in other studies [19,29,31]. However, in this study of pleiotropic DCs, we only focused on indirect interaction, which is mediated by soluble cytokines such as IL-6; the direct cell-cell interaction pattern mediated by surface molecules, including CD80, CD86, CD28 and Toll-like receptors (TLRs), was not studied [19,30,32]. We tested the expression of IFN-γ and IL-17A in different experiment settings, depleted as fold changes compared to saline injection. Both IFN-γ and IL-17A was significantly increased in RRV group at day 7 after RRV infection. In “RRV with Treg transfer” group and in “RRV with Th17 depletion” group, IL-17A and IFN-γ were significantly decreased compared to RRV injection group (S7 Fig). These findings give us hint that Th1 and Th17 might synergistically related to each other. However the interplay between these effector cells still remains unknown [2,17].

By comparing the symptoms of mice after injection of digoxin or Treg cells, we found that survival improvement after Treg cell AT was more satisfactory than after digoxin injection, as depicted in Fig 5A. This evidence linked to the fact that Treg cells may also suppress other effector T cells except for Th17 cells [33].

In summary, our *ex vivo* and *in vivo* studies, using a well-established murine model of BA, provided solid evidence that unopposed overexpansion of Th17 cells due to excess IL-6 levels and impaired suppression of Treg cells participates in the pathogenesis of BA in mice. In this context, Th17 cells, IL-17A or IL-6 may constitute a new molecular candidate that, if blocked, may lead to suppression of autoimmune hepatobiliary injury before or after surgery, alleviating symptoms and promoting long-term survival.

## Supporting Information

S1 Fig IL-17A secretion significantly decreases in liver cells of RRV infected mice with Th17 depletion. Cytokine levels in the supernatant of liver cell suspensions of RRV or saline injected mice were determined by ELISA in mice with or without Th17 depletion by digoxin. Mice were sacrificed on day 7 post infection. The results are reported as the mean ± SEM (n = 15). * *P*<0.05.
(TIF)

S2 Fig Population peak of Th17 and γδT cell occur at different time point after RRV infection in mice. Th17 and γδT cells were harvested from mice liver at different time points and analyzed by FCM. The peak of Th17 (%) appeared on the 10^th^ day and the peak of γδT cells (%) appeared on the 3^rd^ day after injection of RRV. The percentage of Th17 or γδT cells remained relatively low after saline injection.
(TIF)

S3 Fig IL-17A Ab infection increases survival and weight gain in RRV infected mice. **(A)** Weight gain after birth for 3 groups. Mice weights were recorded each day after RRV infection for 16 days, * *P*<0.05 day 5 through day 16. **(B)** Kaplan-Meier survival analysis of mice. 13–17 mice in each group were followed for survival post-infection. *P*<0.001 for RRV *vs* RRV + digoxin.
(TIF)

S4 Fig IL-6 secretion of different cell types cultured ex vivo after isolation from liver. IL-6 levels in the culture medium of macrophages, B cells, CD4+CD25- T cells and cholangiocytes isolated from the livers of BA mice and the control group. * *P*<0.05.
(TIF)

S5 Fig Kinetics of RRV in bile duct and liver tissues in RRV challenged mice with or without IL-17A Ab injection. Kinetics of RRV infection represented by VP6 gene average copies compared to GAPDH expression in RRV and RRV^+^IL17A Ab groups using quantitative PCR. Each data point reflects the average of RRV presence in the bile duct (A) and liver (B). n = 10. No significant difference was noticed between the two groups. * *P*<0.05.
(TIF)

S6 Fig IL-17F mRNA level in liver of mice in different experimental settings. IL-17F mRNA level in RRV challenged mice were tested by RT-PCR at day 4, 7, 10, 14. Samples were collected from in mice of Th17 depleted experiment and Treg adoptive transfer experiment as well. Results are all expressed as fold changes compared to saline injection. * *P*<0.05.
(TIF)

S7 Fig Comparison of IFN-γ and IL-17A expression in RRV infected mice underwent Th17 depletion or Treg transfer. At day 7 post RRV injection, expression of IFN-γ and IL-17A in Th17 depletion and Treg transfer groups were examined, depleted as fold changes compared to saline injection. * *P*<0.05.
(TIF)

S1 Table Primers used in real-time PCR to quantify levels of mRNA expression (DOCX)
