# Supplementary figures and images for: Dendritic Cells Regulate Treg-Th17 Axis in Obstructive Phase of Bile Duct Injury in Murine Biliary Atresia
Source: PLoS One. 2015 Sep 1;10(9):e0136214. doi: 10.1371/journal.pone.0136214 (PMC4556529; doi:10.1371/journal.pone.0136214)

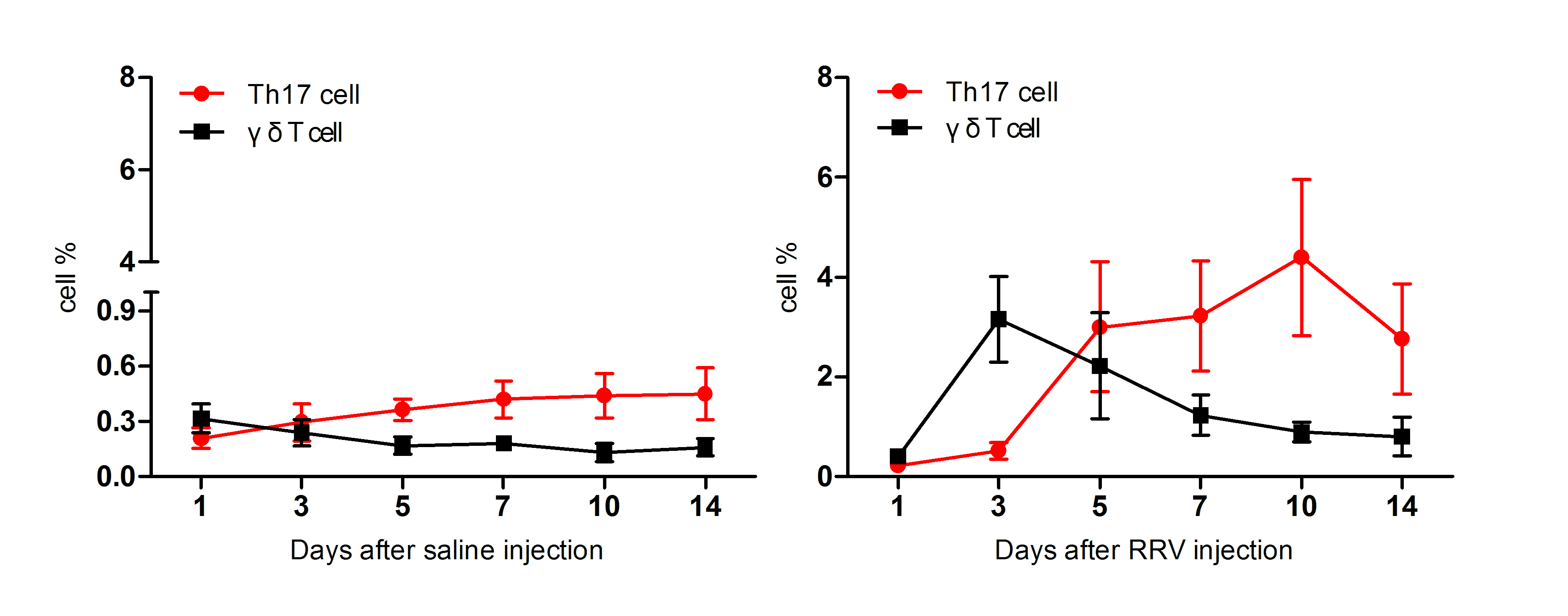

Supplement: S2 Fig — Th17 and γδT cells were harvested from mice liver at different time points and analyzed by FCM. The peak of Th17 (%) appeared on the 10th day and the peak of γδT cells (%) appeared on the 3rd day after injection of RRV. The percentage of Th17 or γδT cells remained relatively low after saline injection. (TIF) [file pone.0136214.s002.tif]

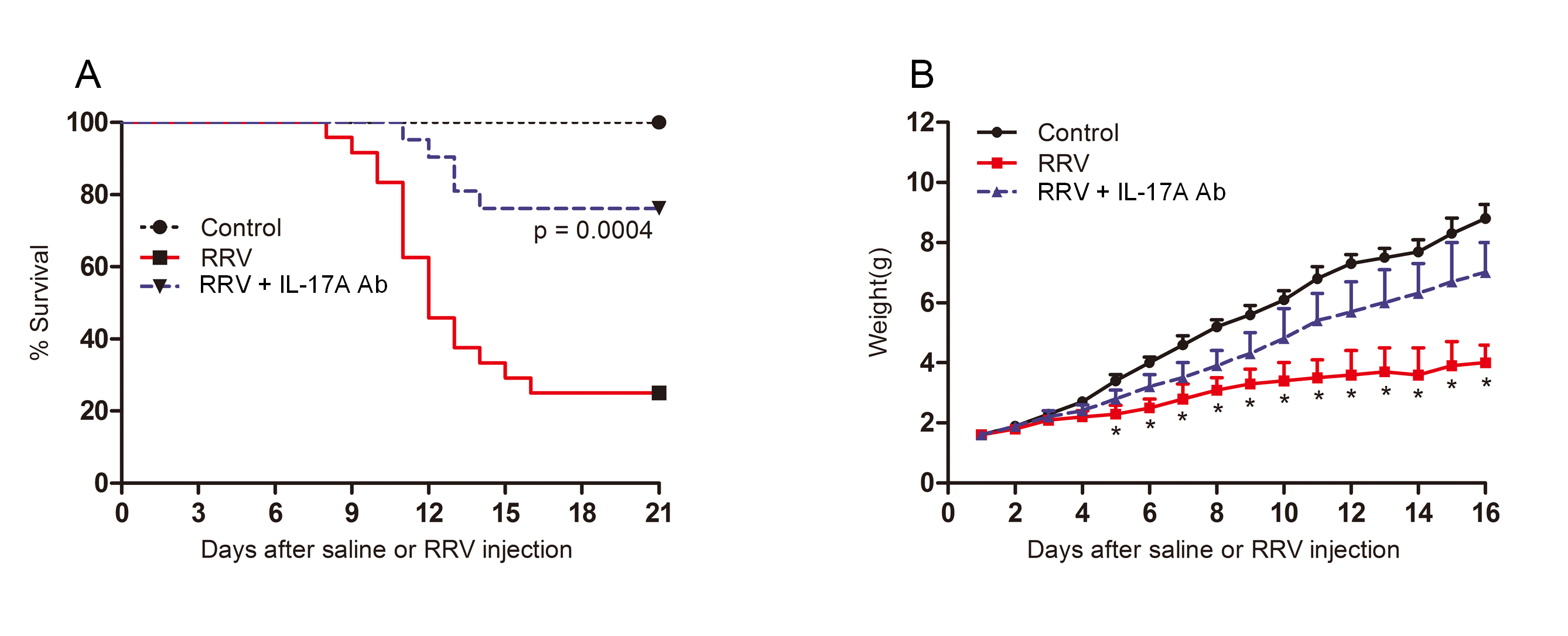

Supplement: S3 Fig — (A) Weight gain after birth for 3 groups. Mice weights were recorded each day after RRV infection for 16 days, * P<0.05 day 5 through day 16. (B) Kaplan-Meier survival analysis of mice. 13–17 mice in each group were followed for survival post-infection. P<0.001 for RRV vs RRV + digoxin. (TIF) [file pone.0136214.s003.tif]

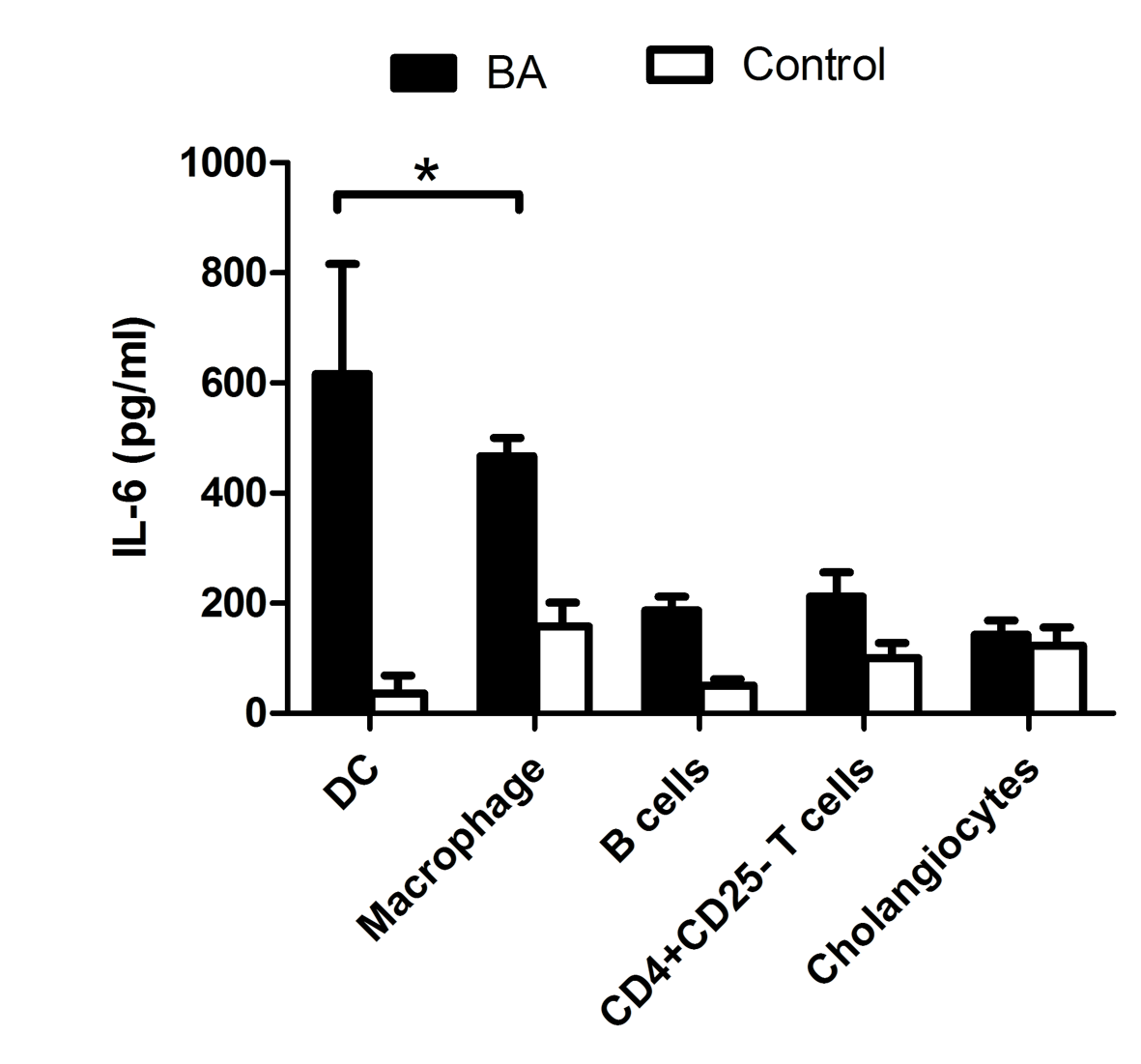

Supplement: S4 Fig — IL-6 levels in the culture medium of macrophages, B cells, CD4+CD25- T cells and cholangiocytes isolated from the livers of BA mice and the control group. * P<0.05. (TIF) [file pone.0136214.s004.tif]
