# Supplementary material for: Dendritic Cells Regulate Treg-Th17 Axis in Obstructive Phase of Bile Duct Injury in Murine Biliary Atresia
Source: PLoS One. 2015 Sep 1;10(9):e0136214. doi: 10.1371/journal.pone.0136214 (PMC4556529; doi:10.1371/journal.pone.0136214)
Supplement: S1 Table — (DOCX) [file pone.0136214.s008.docx]

**S 1 Table.**

| **Primers used in real-time PCR to quantify levels of mRNA expression** | | | |
| --- | --- | --- | --- |
| **Gene** | **Primer Sequences** | **Temp**  **(°C)** | **Size**  **(bp)** |
| *GAPDH* | Forward: 5′-CCCCTTCATTGACCTCAACTAC-3′  Reverse: 5′-CTCGCTCCTGGAAGATGGTGAT-3′ | 59 | 137 |
| *Foxp3* | Forward: 5′-CAGCAGGAGAAAGCGGATACC-3′  Reverse: 5′-GAAGACTTTGAGCAACCTGGAG-3′ | 58 | 204 |
| *IL-17A* | Forward: 5′-CCTCAGACTACCTCAACCGTTC-3′  Reverse: 5′-GGTCCAGCTTTCCCTCCGCATT-3′ | 60 | 135 |
| *ROR-γt* | Forward: 5′-GCAGGAGCAATGGAAGTCG-3′  Reverse: 5′-GATGAGAACCAGGGCCGTGTAG-3′ | 59 | 216 |
| Temp: annealing temperature of primer pair  Size (bp): product size in base pairs | | | |
